# Scarlet Fever

**Published:** 1871-02

**Authors:** 


SCARLET FEVER.—During the twenty-one years from 1848 to 1868, inclusive, there were registered in England and Wales 415,982 deaths from scarlet fever and its allied disease diphtheria. It is estimated that at least 40,000 deaths have occurred in England last year.

